# Perturbing the consistency of auditory feedback in speech

**DOI:** 10.3389/fnhum.2022.905365

**Published:** 2022-08-25

**Authors:** Daniel R. Nault, Takashi Mitsuya, David W. Purcell, Kevin G. Munhall

**Affiliations:** ^1^Department of Psychology, Queen’s University, Kingston, ON, Canada; ^2^School of Communication Sciences and Disorders, Western University, London, ON, Canada; ^3^National Centre for Audiology, Western University, London, ON, Canada

**Keywords:** speech motor control, speech production, auditory feedback, perturbation, consistency, variability, compensation

## Abstract

Sensory information, including auditory feedback, is used by talkers to maintain fluent speech articulation. Current models of speech motor control posit that speakers continually adjust their motor commands based on discrepancies between the sensory predictions made by a forward model and the sensory consequences of their speech movements. Here, in two within-subject design experiments, we used a real-time formant manipulation system to explore how reliant speech articulation is on the accuracy or predictability of auditory feedback information. This involved introducing random formant perturbations during vowel production that varied systematically in their spatial location in formant space (Experiment 1) and temporal consistency (Experiment 2). Our results indicate that, on average, speakers’ responses to auditory feedback manipulations varied based on the relevance and degree of the error that was introduced in the various feedback conditions. In Experiment 1, speakers’ average production was not reliably influenced by random perturbations that were introduced every utterance to the first (F1) and second (F2) formants in various locations of formant space that had an overall average of 0 Hz. However, when perturbations were applied that had a mean of +100 Hz in F1 and −125 Hz in F2, speakers demonstrated reliable compensatory responses that reflected the average magnitude of the applied perturbations. In Experiment 2, speakers did not significantly compensate for perturbations of varying magnitudes that were held constant for one and three trials at a time. Speakers’ average productions did, however, significantly deviate from a control condition when perturbations were held constant for six trials. Within the context of these conditions, our findings provide evidence that the control of speech movements is, at least in part, dependent upon the reliability and stability of the sensory information that it receives over time.

## Introduction

Painted on the window of a café in the Norrmalm district of Stockholm is information to help customers find their way in. Within an arrow pointing to the left is the text, “Entrance 8,47 M”. What makes this signage funny is its precision. Knowing the door’s location to the hundredth of a meter when you are steps away from entering is excessive and it makes passersby smile when they see it. People have an intuitive feel for what information they need and how precise it should be.

Current models of the control of actions include sensory information that is used to coordinate the movements accurately or is needed to maintain the stability of the motor system [see Parrell et al. (2019a) for a review of recent speech models]. Such models include closed-loop processing of sensory information to guide immediate motor responses and predictive algorithms where sensory information is used to tune representations of the effectors and their activities. In both types of sensorimotor control, the required precision of the sensory information and reliability of that information is a part of the control system.

The present paper addresses this issue of the precision of perceptual information for action in a specific context—spoken language. All the papers in this special issue present studies of how the auditory feedback for speech is processed and how it influences the accuracy of talking. The technique that is employed in these papers is the real-time modification of the sounds that talkers produce so that they hear themselves say sounds slightly differently than they actually spoke. Studies have shown that introducing errors in the timing (Mitsuya et al., 2014), amplitude (Heinks-Maldonado and Houde, 2005), pitch (Kawahara, 1995), and spectral details (Houde and Jordan, 1998) of the auditory feedback cause talkers to modify their speech in compensation. The question we are asking here is: How “off” can the feedback be?

The best answer to that question is: it depends. It depends on the vocal parameter. Timing, amplitude, and frequency parameters may be related in spoken language, but they are the purview of different articulatory subsystems, and they convey different communicative information in speech. They are measured in different physical qualities with different units. Thus, there is no simple one-to-one correspondence between their signal ranges or their variabilities.

Here we report studies of variability in speech produced in a very restricted context. Specifically, we present a series of studies of vowel formant feedback produced in repetitive citation format. This choice is determined by factors both pragmatic and strategic. Practically, the custom real-time processor that we use (Purcell and Munhall, 2006) is designed for cued production of a stimulus set where real-time formant tracking is optimized for a particular vowel. Repetitive productions of the same syllable are ideal for this paradigm.

Our strategic reason for using repetitive syllable production is that we aim to understand the operating principles of the most basic speech utterances spoken at a normal rate. By using feedback perturbations on a syllabic unit, we are trying to carry out system identification for speech motor behavior. With controlled conditions, and the subject performing the same task (e.g., moving to the same target), the character of the dynamic system that controls articulation can be uncovered^1^. This is an admittedly reductionist approach, but we believe it serves as important baseline behavior of the much more complex system.

Our focus here will be on trial-to-trial variability within and between subjects. Variability is one of the hallmarks of speech and motor systems generally, and it can be the result of ‘noise’ at many levels in the nervous system (Faisal et al., 2008: cellular, synaptic, sensory, motor, etc.). Such noise can be seen as a challenge for control but is also thought to be beneficial in some circumstances (e.g., in learning and skill acquisition: Dhawale et al., 2017; Sternad, 2018). Here we treat it as a biomarker of the state of the system (Riley and Turvey, 2002) as we assess changes in the predictability of auditory feedback in speech.

Vowel production in both acoustic and articulatory terms shows considerable variability (e.g., Whalen et al., 2018) but variability that is consistent across vowels and correlated for acoustics and articulation. While this variability can change over the course of a day, it is relatively stable across days (Heald and Nusbaum, 2015). Because of these attributes, changes in variability are frequently used as an index of developmental stage (Sosa, 2015) and clinical status (Miller, 1992). We will use this parameter as an index of how the speech system responds to changes in the predictability of auditory feedback.

Studying the predictability of auditory feedback has several important advantages. Experimentally, it is something that can be manipulated in the real-time feedback paradigm. Critically, it is also at the heart of most current computational models of speech, including DIVA (Villacorta et al., 2007), GEPPETO (Patri et al., 2018, 2019), and FACTS (Parrell et al., 2019b). Forward models are proposed to predict the sensory consequences of speaking and adjust future motor commands to the computed discrepancies between model and sensory feedback. Sensorimotor speech control is thought to be inherently predictive.

### The present studies

**FIGURE 1:**
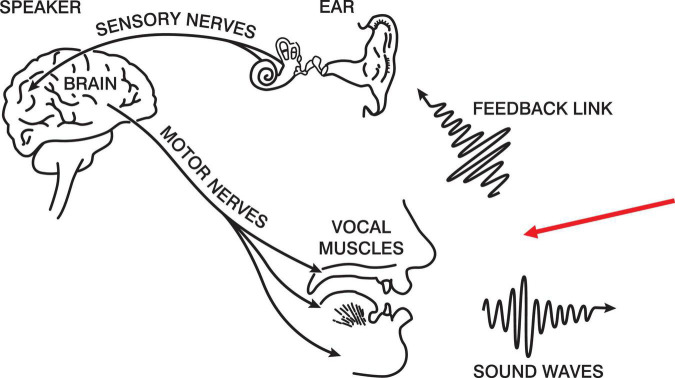
The Speech Chain (Denes and Pinson, 1973).

Below in Figure 1 is a modified version of the production half of Denes and Pinson’s (1973) speech chain. The figure portrays a closed loop between intention and the feedback that talkers hear of their own speech. The red arrow indicates our experimental intervention. Our proposal is that, if subjects are producing naturally-paced syllables^2^, we can manipulate the regularity of the auditory feedback and determine the assiduity of the forward model.

Prior to considering our manipulations, it is useful to reflect on what is known of the boundary conditions of the auditory feedback system. For both temporal and spectral perturbations, there are demonstrated ranges over which subjects respond to change. In formant perturbation studies that increase or ramp the changes by small amounts on successive utterances, subjects do not produce compensations on average until the perturbation is beyond a threshold (Purcell and Munhall, 2006). It is as if there is a tolerance for variation in production and small errors do not require correction. At the other end of the perturbation range, subjects’ compensations increase linearly with steps in the ramp until the perturbations become too large (MacDonald et al., 2010). Compensations in both the first and second formant reach an asymptote and, as perturbations in the experiment continue to increase with each utterance, the compensation starts to decrease. Finally, the auditory feedback system operates optimally with simultaneous feedback and less so with delays (Mitsuya et al., 2017). Mitsuya et al. (2017) showed that with delays decreasing from 100 ms, the compensation grew linearly to simultaneity. This presents a picture of a formant control system that has inherent variability and that operates within a bounded set of conditions. It does not correct changes smaller than a range of about plus or minus 50 Hz. The control system does not make changes specifically tied to large perturbations of more than 250 Hz and compensates most strongly when there are no delays in auditory feedback. Sudden step changes in formant frequency within this span of conditions are compensated over a series of utterances (approximately 10 trials) rather than on the next trial.

Here, we aim to explore, within the scope of these conditions, how reliant speech articulation is on a predictable auditory feedback environment over a sequence of utterances. In the study of visuomotor and force field paradigms for limb movement, manipulations of feedback predictability have advanced Bayesian perspectives on motor adaptation and sensorimotor control (see Krakauer et al., 2019 for a review). The extension of this approach to speech production has been limited. Daliri and Dittman (2019) have addressed these issues in a series of papers. Their work suggests that task relevance and the magnitude of the error influence the magnitude of the observed compensation. Here, we extend this work by applying manipulations to the probability of perturbation and the consistency or range of the errors that speakers hear.

The data presented in this paper stem from two separate experiments, each involving multiple conditions. Experiment 1 was conducted at the University of Western Ontario, while Experiment 2 was conducted at Queen’s University. The raw data are publicly available on OSF here: osf.io/n4pgf.

## Experiment 1

In Experiment 1, we directly manipulated the predictability and, therefore, the variability of the auditory feedback of speakers’ formant frequencies during vowel production. In three experimental conditions, we constrained the auditory feedback speakers received into specific regions of the F1/F2 vowel space. Our aim was to examine the influence of systematic variability in auditory feedback on speakers’ moment-to-moment and average speech production patterns.

We selected three different types of feedback variability that varied in the range of the feedback error introduced, and in the degree of independence of the perturbations to F1 and F2:

1. Randomly and independently perturbing F1 and F2 on each trial over the frequency range that would change the syllable “head” to either “hid” or “had” (F1 ±200 Hz; F2 ±250 Hz) but with an overall mean perturbation of 0 Hz in both formants.
2. The same random perturbations over the same frequency range but only for F1. No perturbation was applied to F2. As in the first condition, the overall mean perturbation was 0 Hz.
3. A more phonetic perturbation that randomly varied the feedback for F1 and F2 on each trial in a coupled manner as if the feedback was being shifted between “head” and “had.” This varied the vowel quality within a small region of the vowel space and smaller region of the acoustic space (F1 +200 Hz; F2 −250 Hz). We used this condition to also test whether introducing a bias to the randomization would influence the behavior of the speech motor system. In this condition, the mean perturbation across trials was F1 = 100 Hz and F2 = −125 Hz.

These feedback perturbations are only a subset of the ways that unpredictability could alter feedback processing in fluent speech. However, they sample distinct modes of noise in speech feedback and will serve to test in a broad way the dependence on similar noise levels in F1 and F2. They also provide an initial test of the effects of the range of perturbation variability.

### Materials and methods

#### Participants

Eighteen female speakers fluent in Canadian English ranging in age from 21 to 30 years of age (*M*_*age*_ = 24.06, *SD*_*age*_ = 2.26) participated in the study. Eight speakers reported being fluent in at least one other language in addition to English. To reduce variability in formant values due to sex differences, only female participants were recruited. All participants had normal audiometric hearing thresholds between 500 and 4,000 Hz (≤20 dB hearing level) and reported having no speech or language impairments. All participants provided written, informed consent prior to participating and all experimental procedures were approved by the Health Sciences Research Ethics Board at Western University.

#### Equipment

The equipment used for Experiment 1 was the same as previously reported in Mitsuya et al. (2017). Participants sat in front of a computer monitor in a sound-attenuated booth (Eckel Industries of Canada, model C2) and wore headphones (Sennheiser HD 265). Their speech was recorded using a portable headset microphone (Shure WH20). The microphone signal was amplified (Tucker-Davis Technologies MA3 microphone amplifier), low-pass filtered with a cut-off frequency of 4,500 Hz (Frequency Devices type 901) and digitized at a sampling rate of 10 kHz. The signal was then filtered in real-time to produce formant feedback perturbations (National Instruments PXI-8106 embedded controller). The processed speech signal was presented back to participants with Sennheiser HD 265 headphones at approximately 80 dBA sound pressure level (SPL) with speech shaped noise (Madsen Itera) of 50 dBA SPL.

#### Acoustic processing

Voicing was detected using a statistical amplitude threshold, and formant manipulations were introduced in real time using an infinite impulse response filter (see Purcell and Munhall, 2006). An iterative Burg algorithm (Orfanidis, 1988) was implemented to estimate formant changes every 900 μs. Formant estimates were then used to calculate filter coefficients. A pair of spectral zeros were used to deemphasize energy present in the existing formant frequency, and a pair of spectral poles were used to emphasize energy present in the new desired formant.

Prior to data collection, talkers were cued to randomly produce six tokens of each English vowel in the/hVd/context (“heed,” “hid,” “hayed,” “head,” “had,” “hawed,” “hoed,” “who’d,” “hood,” and “heard”). This was carried out to estimate a parameter that determined the number of coefficients used in the real-time filtering of the vowels in the experiment. Participants were presented with a visual prompt of each word that remained on a computer screen for 2.5 s (with an inter-stimulus interval of approximately 1.5 s).

Formants were analyzed offline in the same manner as previously reported in Munhall et al. (2009). For each utterance, vowel boundaries of the vowel segment were estimated using an automated process based on the harmonicity of the power spectrum. Vowel boundaries were then inspected by hand and corrected, if necessary. Trials were occasionally removed from the dataset when participants made an error (i.e., pronounced the wrong word, failed to produce the correct vowel, coughed or lip smacked during production). The same algorithm that was used for real-time formant tracking was also used offline to estimate the first three formant frequencies (F1, F2, and F3) for each utterance. Formants were estimated from the middle 40–80% of each vowel’s duration. On the occasion when a formant was incorrectly categorized as another (e.g., F1 was categorized as F2), it was manually corrected by inspecting the utterance with all the “steady state” F1, F2, and F3 estimates marked for that participant.

#### Design and procedure

Prior to the experiment, participants filled out a questionnaire to indicate their native language and current language(s) spoken, and to screen for any known vision, hearing, speech, and language impairments. Each participant also completed a hearing screening test at octave frequencies of 500, 1,000, 2,000, and 4,000 Hz prior to beginning the speech experiment.

**FIGURE 2:**
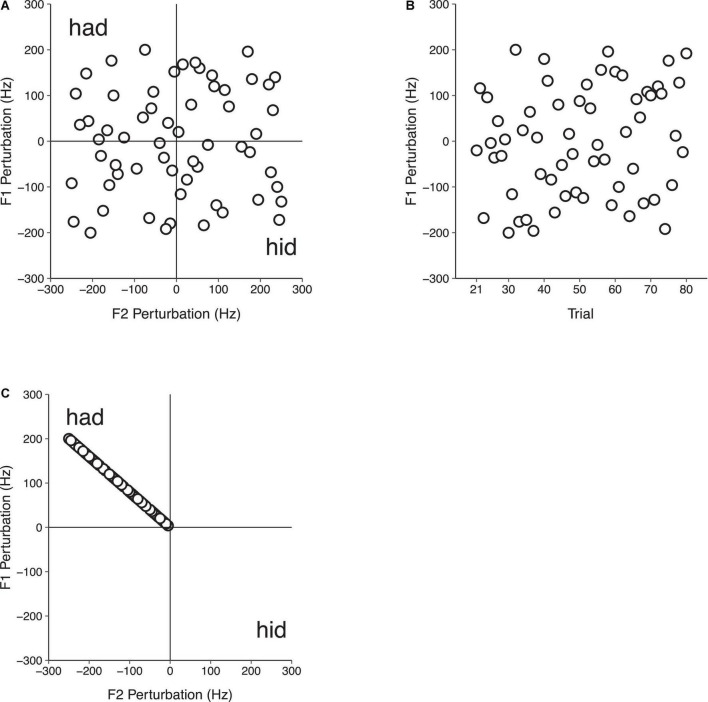
Auditory feedback perturbation values in the Perturbation phase (trials 21–80) of Experiment 1 in the F1/F2 Random Perturbation Condition **(A)**, F1-Only Random Perturbation Condition **(B)**, and F1/F2 Coupled Random Perturbation Condition **(C)**. In the F1/F2 Random Perturbation Condition, perturbation magnitudes were not related. Half of the perturbations were positive, and half were negative. The overall average perturbation value in F1 and F2 was 0 Hz. In the F1-Only Random Perturbation Condition, only F1 was perturbed. An equal number of random positive and negative F1 perturbations were applied, and the overall average perturbation value in F1 was 0 Hz. In the F1/F2 Coupled Random Perturbation Condition, speakers received feedback that was biased toward the vowel/æ/in “had” in F1/F2 space. Random perturbation magnitudes in F1 and F2 were related, with F1 and F2 perturbations being applied in multiples of 4 and –5 Hz, respectively.

Participants sat in a sound-attenuated booth in front of a computer monitor and said the monosyllabic word “head” 140 times in each of three conditions: (1) F1/F2 Random Perturbation Condition, (2) F1-Only Random Perturbation Condition, and (3) F1/F2 Coupled Random Perturbation Condition. In each condition, three successive experimental phases that were not indicated to participants were tested. In the Baseline phase (trials 1–20), participants spoke while receiving natural, unaltered auditory feedback. In the Perturbation phase (trials 21–80), participants’ auditory feedback was manipulated. In the F1/F2 Random Perturbation Condition, this involved randomly perturbing F1 and F2 in multiples of 4 and 5 Hz, respectively, on each trial. The magnitude of the perturbations in F1 and F2 were not related. However, the directions of the perturbations in F1 and F2 were equally distributed. A quarter (i.e., 15) of the perturbations in F1/F2 were both positive (F1 + F2+), both negative (F1 − F2−), and one positive and one negative (F1 + F2−; F1 − F2 +). The overall average perturbation magnitude during the Perturbation phase of the F1/F2 Random Perturbation Condition was 0 Hz in F1 and F2 (see Figure 2A). In the F1-Only Random Perturbation Condition, perturbations were applied in the same way, but only in F1. As in the F1/F2 Random Perturbation Condition, an equal number of positive and negative F1 perturbations were applied during the Perturbation phase (see Figure 2B). In the F1/F2 Coupled Random Perturbation Condition, speakers were presented with perturbations that biased the auditory feedback they received from the vowel /ε/ in “head” toward the vowel/æ/in “had” in F1/F2 space (see Figure 2C). This was achieved by randomly applying positive F1 perturbations in multiples of 4 Hz ranging from +4 to +200 Hz. The average F1 perturbation value was +100 Hz. Perturbation values in F2 were negative and were determined by dividing the value of the F1 perturbation by four and multiplying by negative five. All subjects received the same randomization of perturbations in each condition. The final Return phase (trials 81–140) was the same in all three conditions; participants’ natural unaltered auditory feedback was restored.

The order of each condition was counterbalanced across participants. Before the experiment began, the experimenter instructed participants to speak in their normal conversational voice, and to keep the loudness and pitch of their voice as stable as possible throughout the experiment. To ensure participants returned to baseline speech production after each condition, the experimenter entered the sound booth, and engaged in a short conversation with the participant for a few minutes.

#### Data analysis

The procedure for data analysis involved first eliminating trials 1–5 from the dataset to minimize the impact of subjects’ familiarization with the speech task and with speaking while receiving feedback through headphones. Each speaker’s utterances were then normalized for each condition by subtracting that speaker’s mean Baseline formant frequencies from each of their utterances. This procedure facilitated our ability to compare formant frequencies across speakers. Speakers’ normalized F1 and F2 values were used as the dependent variable in all reported analyses. Descriptive statistics of raw formant values are provided in the Supplementary material.

In both experiments, linear mixed-effects modeling (LMM) was used to examine the influence of condition and phase on speakers’ normalized speech production. Modeling was carried out using the lme4 package (v1.1-27; Bates et al., 2015) in R (R Core Team, 2020). Analyzing our data in this way allowed for the simple handling of missing data. It also allowed us to maximize our control over unexplained variance in formant frequencies among individual speakers by including a random-effects term. For each experiment, two linear mixed-effects models were constructed—one for F1 and one for F2. As per the guidelines set forth by Barr et al. (2013), the random effects structure for each model was kept as maximal as possible based on our experimental design and the satisfaction of model convergence criteria. In each model, this involved including a random intercept for speakers causing non-independence in the data and, if possible, a random slope for each within-unit predictor if there were no convergence errors. If convergence criteria were not satisfied, the random effects structure was simplified by removing the random slope that explained the smallest amount of variance. This process was continued until the random effects model converged (Barr et al., 2013). The random effects structure for each model was determined prior to adding any fixed effects.

In each LMM analysis, we refer to the model with the best fit to the data as the Best Fit Model. In all cases, Best Fit Models were determined using a “backward-fitting” model selection approach (Bates et al., 2015). This involved first testing a model with the maximal random effects structure that satisfied convergence criteria and all fixed effects of interest (i.e., condition, phase, and their interaction term). Fixed effects were then removed one at a time and alternative models were compared for goodness of fit to the data using likelihood ratio tests (LRTs). Two-tailed *p*-values and confidence intervals were estimated using a Wald *t*-distribution with Satterthwaite approximation. The Best Fit Model for each analysis always significantly outperformed all other testable models and satisfied convergence criteria. In cases where significant fixed effects were observed, the emmeans package (v.1.7.0; Lenth, 2019) was used to conduct pairwise comparisons with the Bonferroni correction. In secondary analyses, within-subjects ANOVAs (one for F1, one for F2) were used to examine whether average within-speaker variability (i.e., standard deviation) differed by condition and phase.

We also investigated the possibility of oscillations in compensation throughout the Perturbation phase of each condition. This was achieved by computing an amplitude spectrum for each subject in each condition using the normalized F1 values from the Perturbation phase as time series. The spectra were calculated in MATLAB (2020b) using a discrete Fourier transform with a Hanning window and a sampling rate of one sample per trial. The resulting amplitude spectra had normalized units of frequency (normalized by the sampling rate and reported as cycles per trial) and were averaged across subjects for each condition. If there was a prominent oscillation of F1 values across trials in the Perturbation phase of any condition, it would be expected to appear as a peak in the frequency spectrum. By averaging only the amplitude spectra, between-subject variability in the temporal position of cycles of a potential oscillation across trials in the Perturbation phase will not diminish detection of the oscillation in the average spectrum.

### Results

The primary dataset for Experiment 1 involved a total of 7,290 utterances (18 speakers * 3 conditions * 135 trials = 7,290). Thirty F1 values and 43 F2 values were omitted from the dataset due to issues with formant tracking. The reported results involve normalized formant frequencies. We begin by visually presenting the average normalized results for F1 and F2 in each condition. We then report the results from the Best-Fit Models used to predict normalized speech production in F1 and F2, followed by analyses of average within-speaker variability.

**FIGURE 3:**
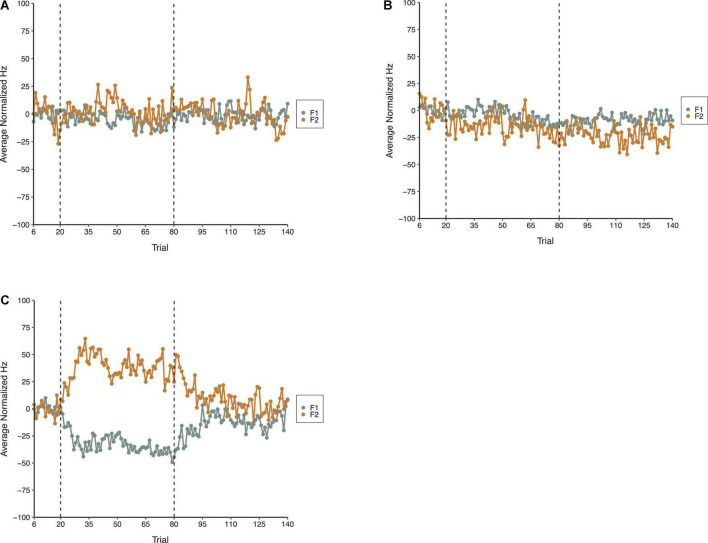
Average normalized F1 (gray) and F2 (gold) speech production values from 18 speakers in the F1/F2 Random Perturbation Condition **(A)**, F1-Only Random Perturbation Condition **(B)**, and F1/F2 Coupled Random Perturbation Condition **(C)** of Experiment 1. From left to right, the dotted lines denote boundaries between the Baseline, Perturbation, and Return phases, respectively.

The average normalized results for F1 and F2 across all three phases of each condition in Experiment 1 are shown in Figure 3. The general pattern apparent in Figure 3 is that the random perturbations with a mean of zero relative formant frequency in the F1/F2 Random Perturbation Condition and F1-Only Random Perturbation Condition had minimal effects on average formant production. In contrast, when the random perturbations had a mean of F1 = 100 Hz and F2 = −125 Hz in the F1/F2 Coupled Random Perturbation Condition, the average compensations resembled those produced in experiments with a step perturbation (e.g., Munhall et al., 2009; MacDonald et al., 2011).

In the LMM analysis of speakers’ normalized F1 speech production values, the Best-Fit Model produced a significantly better fit to the data than a null model that only included the random effects, χ^2^(8) = 505.67, *p* < 0.001. It also significantly outperformed alternative models that only included the fixed effect of Phase [χ^2^(6) = 301.12, *p* < 0.001] or Condition, χ^2^(6) = 492.82, *p* < 0.001. The Best Fit Model was a significantly better fit to the data than another alternative model that did not include the interaction between Condition and Phase, χ^2^(4) = 288.24, *p* < 0.001.

Results from the Best-Fit Model revealed a significant Phase effect. Pairwise comparisons using the Bonferroni correction revealed that speakers’ normalized F1 values were significantly more negative during the Perturbation (*M* = −14.02, *SE* = 2.95) and Return (*M* = −7.64, *SE* = 2.95) phases than they were during the Baseline phase (*M* = −0.04, *SE* = 3.06), all *p*s < 0.001. The main effect of Condition was not significant. However, there was a significant interaction between Condition and Phase. Adjusting for multiple comparisons, pairwise tests showed that there were significant mean differences between the F1 values produced by speakers during the Perturbation phase of the F1/F2 Coupled Random Perturbation Condition (*M* = −32.47, *SE* = 3.64), and the Perturbation phases of the F1/F2 Random Perturbation Condition (*M* = −4.80, *SE* = 4.71) and F1-Only Random Perturbation Condition (*M* = −4.79, *SE* = 3.78), both *p*s < 0.001. In the F1-Only Random Perturbation Condition, speakers’ F1 values were significantly more negative during the Return phase (*M* = −8.88, *SE* = 3.78) than during the Baseline phase (*M* = −0.009, *SE* = 4.03), *p* < 0.001. In the F1/F2 Coupled Random Perturbation Condition, speakers’ F1 values were also significantly more negative during the Return phase (*M* = −13.43, *SE* = 3.64) than during the Baseline phase (*M* = 0.03, *SE* = 3.89), *p* < 0.001. Thus, on average, speakers did not reliably compensate for random F1 perturbations that had a relative overall average of 0 Hz. However, when random F1 perturbations had an average that deviated from zero, speakers demonstrated significant compensatory behavior. In two conditions, speakers’ average F1 production also remained significantly negative as compared to the Baseline phase following the restoration of their natural auditory feedback during the Return phase. A full list of pairwise comparisons and their significance values are provided in the Supplementary material. Best-Fit Model coefficients are shown in Table 1.

**TABLE 1:** Coefficients from the Best-Fit Model used to predict speakers’ normalized F1 values during Experiment 1.

Fixed effects	Estimate (*SE*)	95% CI	*t*-value	*P*-value	Random effects	*SD*
Intercept (F1/F2 condition baseline)	−0.15 (4.92)	[−10.39, 10.08]	−0.03	0.975	*Speaker*	
F1-only condition	0.14 (5.16)	[−10.49, 10.78]	0.03	0.978	Intercept (F1/F2 random condition)	19.71
Linear condition	0.18 (4.80)	[−9.68, 10.05]	0.04	0.970	F1-only condition	19.66
**Perturbation phase**	−**4.65 (1.80)**	**[**−**8.18,**−**1.11]**	−**2.58**	**0.010**	Linear condition	17.94
Return phase	−0.46 (1.80)	[−4.00, 3.07]	−0.26	0.797	*Residual*	26.27
F1-only*perturbation	−0.14 (2.54)	[−5.13, 4.85]	−0.05	0.956		
Linear*perturbation	−**27.85 (2.54)**	**[**−**32.84,**−**22.87]**	−**10.95**	**<0.001**		
F1-only*return	−**8.41 (2.54)**	**[**−**13.39,**−**3.42]**	−**3.30**	**<0.001**		
Linear*return	−**13.00 (2.54)**	**[**−**17.98,**−**8.01]**	−**5.11**	**<0.001**		

Significant effects are bolded. 95% confidence intervals and *p*-values were computed using a Wald *t*-distribution with a Satterthwaite approximation. Number of observations = 7,260; Number of speakers = 18.

The Best-Fit Model predicting speakers’ normalized F2 production was a significantly better fit to the data than a null model that only had the random effects, χ^2^(8) = 373.2, *p* < 0.001. An alternative model that did not have the Condition effect failed to converge. The Best-Fit Model significantly outperformed alternative models that did not have the Phase effect [χ^2^(6) = 354.59, *p* < 0.001], or the interaction between Condition and Phase [χ^2^(4) = 208.91, *p* < 0.001], both *p*s < 0.001.

Results from the Best-Fit Model in F2 revealed that the main effects of Condition and Phase were not significant. However, there was a significant interaction between Condition and Phase. Pairwise comparisons using the Bonferroni correction revealed that, on average, speakers’ F2 production was significantly more positive during the Perturbation phase of the F1/F2 Coupled Random Perturbation Condition (*M* = 38.26, *SE* = 6.99) than during the Perturbation phases of the F1/F2 Random Perturbation Condition (*M* = 3.07, *SE* = 7.51) and the F1-Only Random Perturbation Condition (*M* = −14.86, *SE* = 5.05), both *ps* < 0.001. In the F1-Only Random Perturbation Condition, there were significant mean differences between speakers’ F2 values produced during the Baseline phase (*M* = −0.20, *SE* = 5.56) and Return phase (*M* = −22.93, *SE* = 5.05), and between the Perturbation phase (*M* = −14.86, *SE* = 5.05) and the Return phase, both *p*s < 0.001. In the F1/F2 Coupled Perturbation Condition, speakers’ average F2 production also significantly differed in the Baseline phase (*M* = 0.34, *SE* = 7.36) as compared to the Return phase (*M* = 9.93, *SE* = 6.99; *p* = 0.047), and in the Perturbation phase (*M* = 38.26, *SE* = 6.99) as compared to the Return phase, *p* < 0.001. Hence, as in the F1 model, speakers’ compensatory behavior in F2 was most pronounced during the F1/F2 Coupled Random Perturbation Condition, where average relative perturbation magnitudes deviated from zero. A full list of pairwise comparisons and their significance values are provided in the Supplementary material. Best-Fit Model coefficients for F2 are shown in Table 2.

**TABLE 2:** Coefficients from the Best-Fit Model used to predict speakers’ normalized F2 values during Experiment 1.

Fixed effects	Estimate (*SE*)	95% CI	*t*-value	*P*-value	Random effects	*SD*
Intercept (F1/F2 condition baseline)	−0.03	[−16.36, 16.30]	−0.004	0.997	*Speaker*	
F1-only condition	−0.17	[−18.73, 18.38]	−0.02	0.985	Intercept (F1/F2 random condition)	19.71
Linear condition	0.37	[−15.12, 15.86]	0.05	0.961	F1-Only condition	19.66
Perturbation phase	3.10	[−2.73, 8.93]	1.04	0.297	Linear condition	17.94
Return phase	−0.36	[−6.18, 5.47]	−0.12	0.905	*Residual*	26.27
F1-only*Perturbation	−**17.76**	**[**−**26.01,**−**9.52]**	−**4.22**	** < 0.001**		
Linear*Perturbation	**34.81**	**[26.56, 43.06]**	**8.27**	**<0.001**		
F1-only*Return	−**22.37**	**[**−**30.62,**−**14.13]**	−**5.32**	** < 0.001**		
Linear*Return	**9.94**	**[1.69, 18.19]**	**2.36**	**0.018**		

Significant effects are bolded. 95% confidence intervals and *p*-values were computed using a Wald *t*-distribution with a Satterthwaite approximation. Number of observations = 7,247; Number of speakers = 18.

**FIGURE 4:**
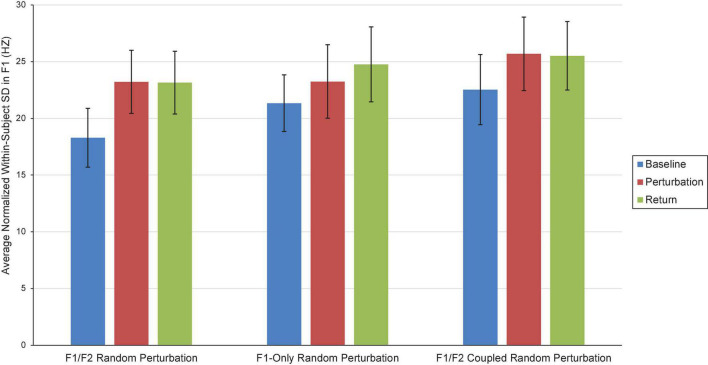
Average normalized F1 within-subject variability (i.e., SD) in the Baseline (blue), Perturbation (red), and Return (green) phases of the F1/F2 Random Perturbation Condition, F1-Only Random Perturbation Condition, and F1/F2 Coupled Random Perturbation Condition in Experiment 1. Error bars represent 95% confidence intervals.

Two repeated-measures ANOVAs (one for F1, one for F2) were carried out to examine the influence of Condition (F1/F2 Random Perturbation, F1-Only Random Perturbation, F1/F2 Coupled Random Perturbation) and Phase (Baseline, Perturbation, and Return) on average within-subject speech production variability (i.e., standard deviation; SD). In the F1 model, the Phase effect violated the sphericity assumption, Mauchly’s Test of Sphericity, *p* = 0.002. The Greenhouse–Geisser correction was thus used to make decisions about the statistical significance of this effect. The main effect of Condition was not significant at the 0.05 level, *F*(2,34) = 3.14, *p* = 0.056, η_p_ = 0.156. However, there was a significant Phase effect, *F*(1.30,22.09) = 5.20, *p* = 0.025, η_p_ = 0.234. Follow-up comparisons revealed that, on average, speakers were significantly less variable in F1 during the Baseline phase (*M* = 20.72; *SE* = 0.984) than they were during the Perturbation (*M* = 24.05, *SE* = 1.21) and Return (*M* = 24.47, *SE* = 1.27) phases, both *ps* < 0.029. The difference in within-speaker F1 variability in the Perturbation and Return phases was not significant, *p* = 0.552. The interaction between Condition and Phase was also not significant, *F*(4,68) = 12.52, η_p_ = 0.038, *p* = 0.609. Average within-subject variability in Experiment 1 is shown in Figure 4.

There were no significant effects in the F2 model. Within-speaker standard deviation in F2 did not significantly differ by Condition [*F*(2,34) = 0.819, *p* = 0.450, η_p_ = 0.046] or Phase, *F*(2,34) = 52.92, *p* = 0.323, η_p_ = 0.064. The interaction between Condition and Phase was also not significant, *F*(2.5,42.51) = 0.608, *p* = 0.585, η_p_ = 0.035.

**FIGURE 5:**
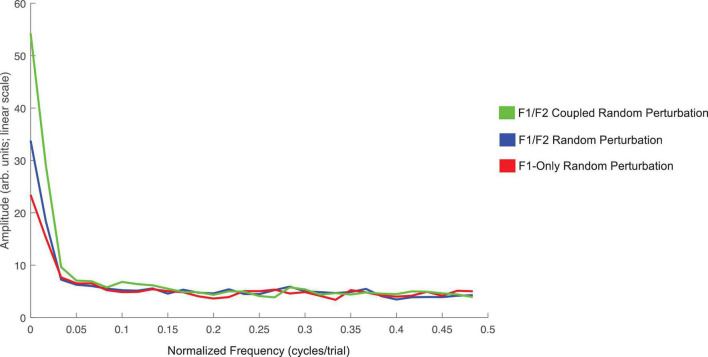
Average amplitude spectra across all 18 speakers for the F1/F2 Coupled Random Perturbation Condition (green), F1/F2 Random Perturbation Condition (blue), and F1-Only Random Perturbation Condition (red) in Experiment 1. The left-most frequency bin of 0 cycles/trial represents the DC-offset. It reflects the mean change in normalized F1 values in the Perturbation phase of each condition as compared to Baseline. Instances of peaks in amplitude at higher frequencies would represent prominent oscillation of F1 values across trials in the Perturbation phase. The spectra were created using a discrete Fourier transform with a Hanning window and sampling frequency set to one sample per trial.

The average amplitude spectrums computed to examine oscillations in speakers’ F1 compensatory behavior throughout the Perturbation phase of each condition in Experiment 1 are presented in Figure 5. The frequency zero represents the DC-offset and reflects the mean change in normalized F1 values in the Perturbation phase relative to the Baseline phase. As can be seen, the mean amplitude at zero cycles/trial for the F1/F2 Coupled Random Perturbation Condition is numerically larger than the other two conditions. This is consistent with the LMM results above. At higher frequencies, all three conditions display low amplitudes and are intermingled, indicating that there were no prominent oscillations of F1 within the Perturbation phase of any condition.

### Discussion

The massive unpredictability of the F1/F2 Random Perturbation Condition and F1-Only Random Perturbation Condition had minimal effects on the formant production characteristics. Variability in the Perturbation and Return phases increased from baseline but only modestly and did so in similar fashions for all three experimental conditions equally for Perturbation and Return phases. While this might be due to the unpredictability of the feedback, our design in these studies does not permit this explanation to be distinguished from a generalized increase in production variance with the extended repetition of the same syllable. This will be examined in Experiment 2.

The average data showed two surprising patterns. First, both the F1/F2 Random Perturbation Condition and the F1-Only Random Perturbation Condition essentially remained at baseline levels. The second surprising result was that the F1/F2 Coupled Random Perturbation Condition, which perturbed the feedback randomly between 0 and +200/−250 Hz (F1/F2) with a mean of +100/−125 Hz (F1/F2), yielded results consistent with a static perturbation of +100/−125 Hz. The observed compensations are approximately 40–50% of the perturbation magnitude, which is consistent with many studies who have used a step perturbation (e.g., Munhall et al., 2009; MacDonald et al., 2011). The results suggest that the compensatory system is integrating feedback error over a sequence of utterances and thus, showing a sensitivity to an average error. In Experiment 2, the temporal consistency of the perturbations will be manipulated to explore the nature of this integration of feedback error. A step perturbation will also be tested to compare the relative consistency of compensation to a static perturbation versus a variable one such as tested here.

## Experiment 2

Our aim in this experiment was to examine whether the feedback system would show greater responsiveness to perturbations held constant for longer periods of time. Such findings would allow us to carry out a preliminary test of the temporal span over which the feedback integrates error information. This experiment also included a non-perturbation control condition where the feedback was held constant, and a step perturbation condition in which feedback was shifted from “head” to “had” during the Perturbation phase.

### Materials and methods

The acoustic processing methods used for Experiment 2 were the same as reported above for Experiment 1. The design and procedure for Experiment 2 was similar to Experiment 1. The equipment was functionally similar to Experiment 1. As such, only differences will be described.

#### Participants

Twenty-two female speakers fluent in Canadian English who did not participate in Experiment 1 were recruited to participate in the study. Two participants were removed from the dataset due to technical issues with the formant perturbation system. The remaining 20 participants ranged in age from 19 to 32 years of age (*M*_*age*_ = 22.35; *SD*_*age*_ = 2.74) and reported having no speech or language impairments. Fourteen speakers reported being fluent in at least one other language in addition to English. All participants had normal audiometric hearing thresholds between 500 and 4,000 Hz (≤20 dB hearing level) and provided their informed consent prior to participating. All experimental procedures were approved by the General Research Ethics Board at Queen’s University.

#### Equipment

The equipment used for Experiment 2 was the same as previously reported in Nault and Munhall (2020). Participants sat in a different sound attenuated booth (Industrial Acoustic Co. model 1201a), and a different controller was used to produce formant shifts in real-time (National Instruments PXI-8176 embedded controller) than in Experiment 1. All other equipment was functionally the same as reported above for Experiment 1.

#### Design and procedure

**FIGURE 6:**
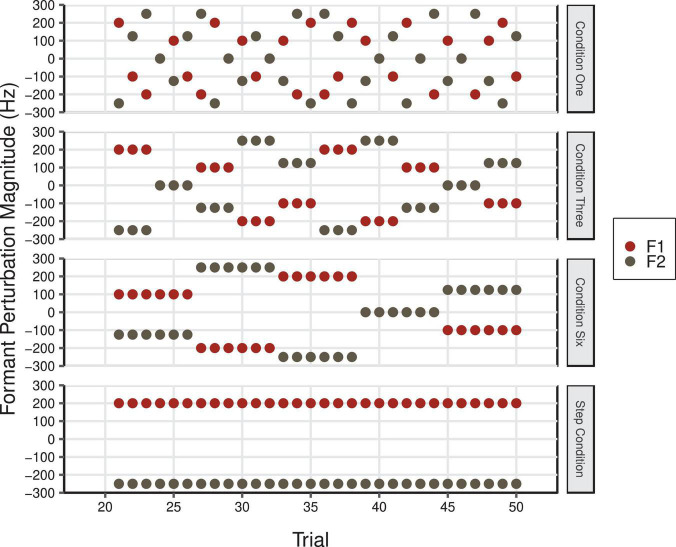
F1 (red) and F2 (gray) perturbation values in Hz during the Perturbation phase of Condition One, Condition Three, Condition Six, and the Step Condition of Experiment 2. The overall average F1 and F2 perturbation values in Condition One, Three, and Six was 0 Hz (F1 min = –200 Hz, F1 max = 200 Hz; F2 min = –250 Hz, F2 max = 250 Hz).

Participants were asked to vocally produce the word “head” 80 times in five different conditions (Control, One, Three, Six, and Step conditions). In the Control Condition, participants received normal, unaltered auditory feedback for all 80 trials. In the four experimental conditions, there were three continuous phases that were not indicated to participants. During the Baseline phase (trials 1–20), speakers received normal, unaltered auditory feedback. Speakers’ auditory feedback was then manipulated during the Perturbation phase (trials 21–50). In conditions One, Three, and Six, perturbations were applied in F1 and F2 with varying levels of temporal predictability (see Figure 6). As in the F1/F2 Coupled Random Feedback Condition in Experiment 1, the feedback perturbations for F1 and F2 were proportional in frequency. Thus, the feedback participants received varied in a linear fashion between the vowel/I/in “hid” to/æ/in “had” in F1/F2 space (see Figure 2C). In Condition One, a different perturbation was introduced on each trial. In Conditions Three and Six, perturbations were held constant for three and six trials, respectively. In all three conditions, the overall average of the F1 and F2 perturbation values was 0 Hz. During the Perturbation phase of the Step Condition, F1 and F2 perturbations of 200 and −250 Hz, respectively, were maintained for 30 trials (see Figure 2B). This is a standard perturbation often used in auditory feedback perturbation studies and it produces a shift across the vowel category boundary from /ε/ to/æ/. In all conditions, participants’ natural auditory feedback was restored during the Return phase (trials 51–80). The order of conditions was counterbalanced across participants.

In between each condition, the experimenter entered the sound booth, and engaged in a few minutes of conversation with each participant. Participants were also asked to read “The Grandfather Passage” (Van Riper, 1963; Darley et al., 1975) aloud. This seminal 132-word passage is often used in clinical settings to elicit oral reading samples and to assess speech motor functioning and speech intelligibility (e.g., De Bodt et al., 2002) due to its semantic and syntactic complexity and diverse range of English phonemes. It was used in the current experiment to encourage speakers to return to baseline vowel production.

### Results

The primary dataset for Experiment 2 included a total of 7,500 utterances (20 speakers * 5 conditions * 75 trials = 7,500). Issues with formant tracking led to the removal of 253 formant values (62 in F1; 191 in F2) from the final dataset. As in Experiment 1, we removed trials 1–5 from the dataset to reduce any possible influence on speech production of task familiarization and speaking while receiving feedback through headphones. We begin by providing a figure of the average normalized results for F1 and F2 in each condition. We then provide results from the Best Fit Models used to predict normalized speech production in F1 and F2. We also report results from within-subjects ANOVAs used to examine within-subject variability. We conclude our Results section with a visual depiction of the average amplitude spectra that were computed to examine oscillations in F1 compensatory behavior throughout the Perturbation phase of each condition.

**FIGURE 7:**
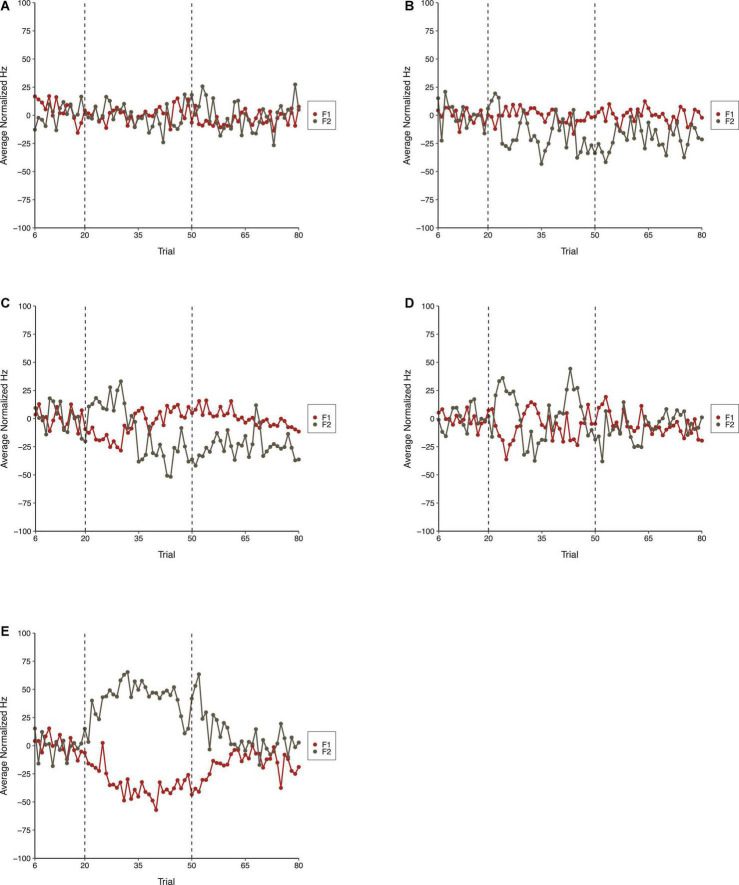
Average normalized F1 (red) and F2 (gray) speech production values in the Control Condition **(A)**, Condition One **(B)**, Condition Three **(C)**, Condition Six **(D)**, and the Step Condition **(E)** of Experiment 2. From left to right, dotted lines denote boundaries between the Baseline, Perturbation, and Return phases, respectively.

The average normalized results for F1 and F2 across all three phases of each condition in Experiment 2 are shown in Figure 7. As shown, the Step Condition, on average, differed from all other conditions during the Perturbation phase. The Perturbation phase of Condition Six differed from the Control Condition indicating that sequential consistency of perturbations was required for compensatory behavior. The results for F2 were similar to F1. On average, the Step Condition produced more robust compensations than any of the other conditions. Compensatory behavior was, on average, more evident in Condition Six than it was during the Control Condition, which suggests that the consistency of perturbations across trials was important for compensation. The F2 results were generally more variable than those observed for F1.

The Best-Fit Model used to predict speakers’ normalized F1 production values in Experiment 2 produced the best fit to the data and included a maximal random-effects structure with random intercepts for speakers. Including random slopes for condition and phase led to model convergence errors. The Best Fit-Model also included the fixed effects of Condition, Phase, and their interaction term. The Best-Fit Model significantly outperformed a null model that only included the maximal random-effects structure, χ2(14) = 499.25, *p* < 0.001, as well as alternative models that only included the fixed effect of Condition [χ2(10) = 200.43, *p* < 0.001] or Phase, χ2(12) = 429.23, *p* < 0.001. The Best-Fit Model was also a significantly better fit to the data than an alternative model that did not have the interaction term, χ2(8) = 127.85, *p* < 0.001.

Results from the Best-Fit Model indicated that there was a significant Phase effect. Pairwise comparisons using the Bonferroni correction indicated that speakers’ normalized F1 values were significantly more negative during the Perturbation phase (*M* = −9.18, *SE* = 2.43) than during the Return phase (*M* = −4.99, *SE* = 2.43) and Baseline phase (*M* = 1.00, *SE* = 2.52), all *p*s < 0.001. The main effect of Condition was not significant. However, there was a significant interaction between Condition and Phase, which was mainly qualified by significant differences between phases of the Step Condition and phases of all other conditions. Notably, speakers’ normalized F1 values were significantly more negative during the Perturbation phase of the Step Condition (*M* = −34.34, *SE* = 2.78) than they were during the Perturbation phases of the Control Condition (*M* = 0.89, *SE* = 2.78), Condition One (*M* = −0.44, *SE* = 2.78), Condition Three (*M* = −5.24, *SE* = 2.78) and Condition Six (*M* = −6.79, *SE* = 2.78), all *p*s < 0.001. Speakers’ mean F1 values were also significantly more negative during the Perturbation phase of Condition Six than they were during the Perturbation phase of the Control Condition, *p* = 0.039. Pairwise differences between the Perturbation phases of all other conditions were not significant. Speakers’ mean F1 values produced during the Return phase of the Step Condition (*M* = −16.40, *SE* = 2.78) were also significantly more negative than those produced during the Return phase of all other conditions, all *p*s < 0.001. A full list of pairwise comparisons is provided in the Supplementary material. Best-Fit Model coefficients are shown in Table 3.

**TABLE 3:** Coefficients from the Best-Fit Model used to predict speakers’ normalized F1 values during Experiment 2.

Fixed effects	Estimate (*SE*)	95% CI	*t*-value	*P*-value	Random effects	*SD*
Intercept (control baseline)	5.34 (3.19)	[−1.03, 11.71]	1.68	0.099	*Speaker* (intercept)	10.41
Condition one	−5.34 (3.06)	[−11.35, 0.66]	−1.74	0.081	*Residual*	37.29
Condition three	−5.33 (3.06)	[−11.34, 0.67]	−1.74	0.082		
Condition six	−5.52 (3.07)	[−11.53, 0.49]	−1.80	0.072		
Step condition	−5.49 (3.08)	[−11.52, 0.54]	−1.79	0.074		
Perturbation phase	−4.45 (2.66)	[−9.66, 0.76]	−1.68	0.094		
**Return phase**	−**8.95 (2.66)**	**[**−**14.16,**−**3.74]**	−**3.37**	**<0.001**		
One*Perturbation	4.02 (3.75)	[−3.33, 11.36]	1.07	0.284		
Three*Perturbation	−0.80 (3.75)	[−8.15, 6.55]	−0.21	0.831		
Six*Perturbation	−2.16 (3.75)	[−9.51, 5.19]	−0.58	0.564		
Step*Perturbation	−**29.73 (3.76)**	**[**−**37.10,**−**22.37]**	−**7.91**	**<0.001**		
One*Return	**9.04 (3.75)**	**[1.69, 16.38]**	**2.41**	**0.016**		
Three*Return	**9.32 (3.75)**	**[1.97, 16.67]**	**2.49**	**0.013**		
Six*Return	3.75 (3.75)	[−3.61, 11.11]	1.00	0.318		
Step*Return	−7.30 (3.76)	[−14.67, 0.07]	−1.94	**0.052**		

95% confidence intervals and *p*-values computed using a Wald *t*-distribution with a Satterthwaite approximation. Significant effects are bolded. Number of observations = 7,438; Number of speakers = 20.

The Best-Fit Model used to predict speakers’ normalized F2 productions included a maximal random effects structure with random intercepts for speakers. It also included fixed effects of Condition, Phase, and their interaction term. The Best-Fit Model significantly outperformed a null model that only included the maximal random-effects structure, χ2(14) = 584.43, *p* < 0.001. It was also a significantly better fit to the data than alternative models that only included the fixed effect of Condition [χ2(10) = 222.54, *p* < 0.001] or Phase, χ2(12) = 520.20, *p* < 0.001. The Best-Fit Model significantly outperformed an alternative model that did not include the interaction between Condition and Phase, χ2(8) = 156.21, *p* < 0.001.

In the Best Fit Model for F2, the main effects of Condition and Phase were not significant. However, there was a significant interaction between these effects. As in the F1 model, the interaction was mainly explained by significant differences between phases of the Step Condition and phases of all other conditions. Importantly, speakers’ average F2 values were significantly more positive during the Perturbation phase of the Step Condition (*M* = 42.47, *SE* = 3.90) than they were during the Perturbation phases of the Control Condition (*M* = −0.25, *SE* = 3.91), Condition One (*M* = −16.62, *SE* = 3.92), Condition Three (*M* = −10.95, *SE* = 3.91), and Condition Six (*M* = 2.72, *M* = 3.91), all *ps* < 0.001. Speakers’ mean F2 values were significantly more negative during the Perturbation phase of Condition One than they were during the Perturbation phases of the Control Condition and Condition Six, both *p*s < 0.001. Speakers’ mean F2 values were significantly more positive during the Perturbation phase of Condition Six than they were during the Perturbation phase of Condition Three, *p* = 0.004. As in the F1 model, there were also a number of significant mean differences between formant values produced during the Return phases of different conditions. A full list of pairwise comparisons is provided in the Supplementary material. Best-Fit Model coefficients for F2 are presented in Table 4.

**TABLE 4:** Coefficients from the Best-Fit Model used to predict speakers’ normalized F2 values during Experiment 2.

Fixed effects	Estimate (*SE*)	95% CI	*t*-value	*P*-value	Random effects	*SD*
Intercept (control baseline)	1.35 (4.55)	[−7.72, 10.41]	0.30	0.768	*Speaker* (intercept)	197.1
Condition one	−1.23 (4.65)	[−10.34, 7.88]	−0.26	0.791	*Residual*	3163.3
Condition three	−1.37 (4.67)	[−10.53, 7.79]	−0.29	0.769		
Condition six	−1.29 (4.66)	[−10.43, 7.85]	−0.28	0.782		
Step condition	−1.54 (4.67)	[−10.69, 7.61]	−0.33	0.742		
Perturbation phase	−1.60 (4.03)	[−9.51, 6.31]	−0.40	0.692		
Return phase	−1.86 (4.04)	[−9.78, 6.07]	−0.46	0.646		
One*Perturbation	−**15.14 (5.70)**	**[**−**26.31,**−**3.97]**	−**2.66**	**0.008**		
Three*Perturbation	−9.33 (5.71)	[−20.53, 1.87]	−1.63	0.103		
Six*Perturbation	4.26 (5.71)	[−6.93, 15.44]	0.75	0.456		
Step*Perturbation	**44.25 (5.70)**	**[33.07, 55.43]**	**7.76**	**<0.001**		
One*Return	−**17.89 (5.69)**	**[**−**29.05,**−**6.73]**	−**3.14**	**0.002**		
Three*Return	−**22.14 (5.72)**	**[**−**33.35,**−**10.94]**	−**3.87**	**<0.001**		
Six*Return	−4.99 (5.71)	[−16.18, 6.21]	−0.87	0.383		
Step*Return	**11.34 (5.71)**	**[0.14, 22.54]**	**1.98**	**0.047**		

Significant effects are bolded. Number of observations = 7,309; Number of speakers = 20.

Two repeated-measures ANOVAs (one for F1, one for F2) were conducted to examine whether within-speaker speech production variability (i.e., SD) differed by Condition (Control, One, Three, Six, and Step) and Phase (Baseline, Perturbation, and Return). One outlier in F1 that was more than three standard deviations from the mean was Winsorized and replaced with the next highest value in the dataset. The F1 model revealed that speakers’ mean speech production variability did not significantly differ by Condition, *F*(4,76) = 0.631, *p* = 0.642, η_p_ = 0.032. However, there was a significant main effect of Phase, *F*(2,38) = 4.85, *p* = 0.013, η_p_ = 0.203. Pairwise comparisons showed that speakers’ F1 productions were significantly more variable during the Perturbation phase (*M*_*SD*_ = 31.64) than they were during the Baseline phase (*M*_*SD*_ = 28.47), *p* = 0.018. There were no statistically significant differences in within-speaker variability between the Baseline and Return (*M*_*SD*_ = 29.75) phases (*p* = 0.172), nor between the Perturbation and Return phases, *p* = 0.052. The interaction between Condition and Phase was only marginally significant, *F*(8,152) = 2.00, *p* = 0.050, η_p_ = 0.095. Using the Bonferroni correction to adjust for multiple comparisons, it was determined that none of the interaction comparisons were significant, all *p*s > 0.059. Notably, there was no significant difference in within-subject variability between the Baseline (*M*_*SD*_ = 30.42), Perturbation (*M*_*SD*_ = 29.16), and Return (*M*_*SD*_ = 28.65) phases of the Control Condition, all *ps* > 0.05.

**FIGURE 8:**
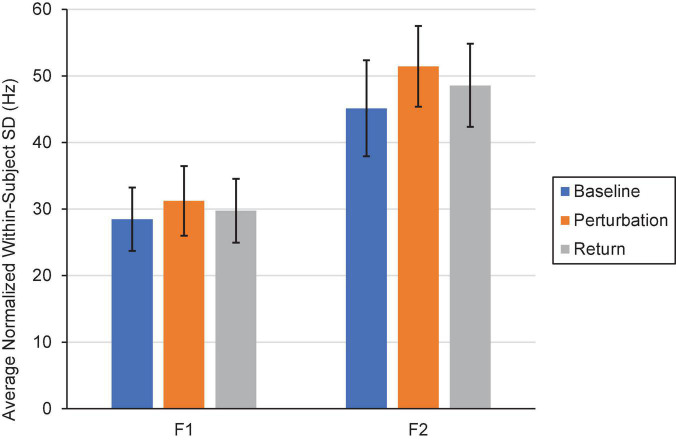
Average normalized F1 and F2 within-subject variability (i.e., SD) in the Baseline (blue), Perturbation (orange), and Return (gray) phases of Experiment 2. Error bars represent 95% confidence intervals.

In the F2 model, the within-subjects effect of Condition and the interaction between Condition and Phase violated the sphericity assumption, Mauchly’s Test of Sphericity, *p*s < 0.05. The Greenhouse-Geisser correction was thus used in making decisions about significance. As in the F1 model, the main effect of Condition was not significant, *F*(2.68,50.96) = 0.499, *p* = 0.664, η_p_ = 0.026. However, there was a significant main effect of Phase, *F*(1.58,30.09) = 5.31, *p* = 0.016, η_p_ = 0.218. Follow-up comparisons revealed that speakers were significantly more variable in F2 during the Perturbation phase (*M*_*SD*_ = 51.44) than they were during the Baseline phase (*M_*SD*_* = 45.12), *p* = 0.012. Within-speaker production variability did not significantly differ between the Baseline and Return (*M*_*SD*_ = 48.59) phases (*p* = 0.106), nor between the Perturbation and Return phases, *p* = 0.054. The interaction between Condition and Phase was not significant, *F*(4.70,89.22) = 2.25, *p* = 0.060, η_p_ = 0.106. A visual depiction of the Phase effect in F1 and F2 is shown in Figure 8.

**FIGURE 9:**
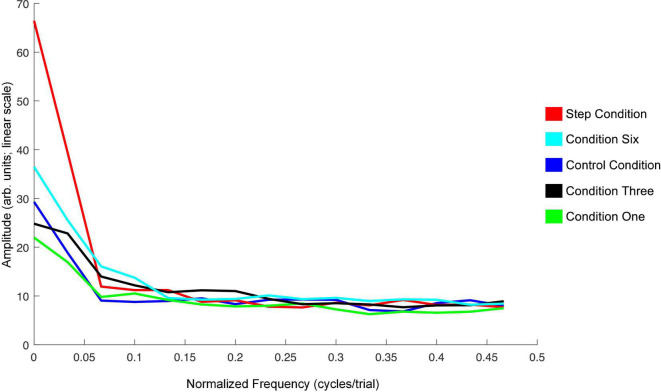
Average amplitude spectra across all 20 speakers for the Step Condition (red), Condition Six (cyan), Control Condition (blue), Condition Three (black), and Condition One (green) in Experiment 2. The left-most frequency bin of 0 cycles/trial represents the DC-offset. It reflects the mean change in normalized F1 values in the Perturbation phase of each condition as compared to Baseline. Instances of peaks in amplitude at higher frequencies would represent prominent oscillation of F1 values across trials in the Perturbation phase. The spectra were created using a discrete Fourier transform with a Hanning window and sampling frequency set to one sample per trial.

The spectra shown in Figure 9 summarize the findings for F1 in Experiment 2. The DC-offset (seen at frequency 0 cycles/trial) shows the only major difference. The Step Condition is larger than the other conditions at this frequency. Condition Six is trending in the same direction. Otherwise, across conditions, there are no differences at higher frequencies in the spectra.

One possible explanation for compensation being significantly more pronounced in the Perturbation phase of the Step Condition and Condition Six than in Condition One and Condition Three is that the feedback error was held constant for a greater number of trials in these two conditions and thus, the error correction system was responding to more stable and predictable conditions.

We computed a series of bivariate correlations between F1/F2 perturbation values that were applied in the Perturbation phase of Condition One, Condition Three, and Condition Six and average normalized F1/F2 production values across all subjects from these three conditions^3^. Correlations could not be computed for the Step or Control Conditions due to the F1/F2 perturbation values being held constant throughout the entire Perturbation phases. Correlations were computed at four lags: zero (simultaneous), one, three, and five trials. Our reasoning was that a comparison between simultaneous and time-lagged correlations would provide insights into whether the error correction system was operating instantaneously, or whether it was integrating information over time.

**FIGURE 10:**
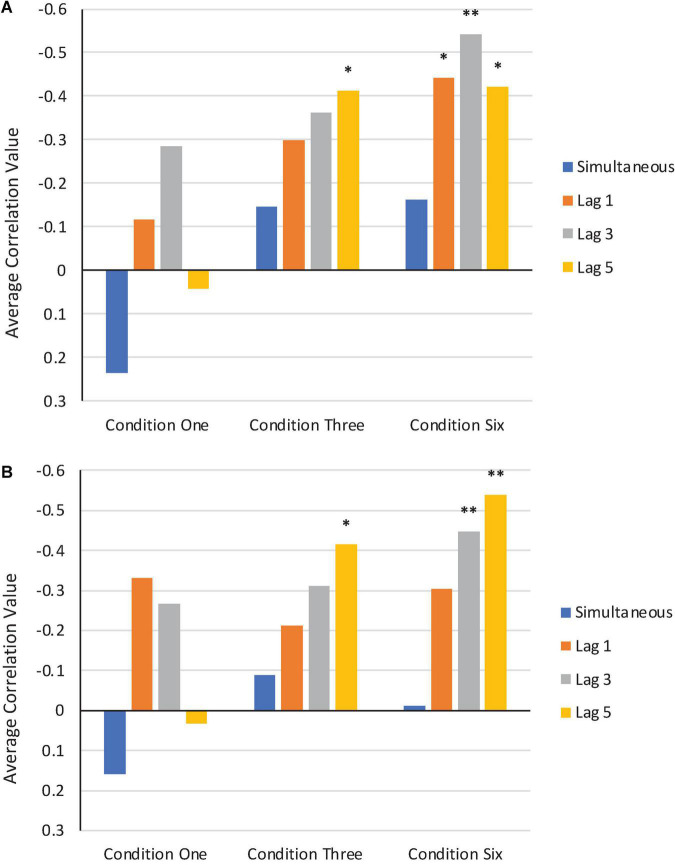
Average bivariate correlations in F1 **(A)** and F2 **(B)** between perturbation values applied in the Perturbation phase of Condition One, Condition Three, and Condition Six and participants’ normalized production values. Simultaneous correlations are shown in blue. Correlations at trial lags of one, three, and five are shown in orange, gray, and yellow, respectively. *Correlation is significant at the 0.05 level. **Correlation is significant at the 0.01 level.

A visual depiction of the average results from the bivariate correlations in F1 and F2 are shown in Figure 10. More negative correlation values indicate stronger compensatory responses.

As can be seen in each condition, the average simultaneous correlation values are much lower (i.e., closer to zero or more positive) than the average lag correlation values. This is particularly the case in Condition Three and Condition Six, where the feedback perturbations were applied in a more consistent and stable manner during the Perturbation phase.

### Discussion

As in Experiment 1, only the introduction of perturbations that consistently deviated from baseline in direction and magnitude produced significant shifts across the Perturbation phase. The step change compensations resembled those observed in other studies that introduced such perturbations (e.g., Munhall et al., 2009; MacDonald et al., 2011). The different length of perturbations (1, 3, and 6 trials) did not significantly differ from each other, although the Six Condition was significantly different from the Control Condition. This finding is consistent with the idea that feedback deviations are compensated incrementally over trials and that six trials is within the span that is required for compensation to develop whereas one and three trials are too short for systematic change to develop in response to perceived errors. The lag correlation findings are consistent with this idea of a span of compensation.

For both F1 and F2, variability increased in the Perturbation phases of all conditions, and this was particularly true for F2. This finding contrasts with other studies that have not shown an increase in variability in perturbation phases (e.g., Nault and Munhall, 2020). Notably, the increase in within-subject variability during the Perturbation phase does not appear to be due to participant fatigue from being asked to say the same word repeatedly, as variability in F1 and F2 did not significantly differ in the three phases of the Control Condition. Rather, the increase in variability appears to be due to the unpredictability of the feedback in the experimental conditions.

## General discussion

The experiments presented here are part of a broad literature in speech, limb, and eye movements that examine a subtype of motor learning called adaptation. Adaptive responses are designed to maintain the accuracy and stability of movements that are already learned when environmental conditions change, or when sensory perception is noisy. Adaptation is thus usually studied in paradigms that focus on reducing error following some form of perturbation. While compensatory response to auditory feedback perturbations is well documented, here we examined the speech compensation when the sensory feedback returned unpredictable errors.

Across a number of different interpretations of randomness in feedback, our results indicate that the use of auditory feedback in speech motor control is governed by the relevance of the feedback. Talkers acted, on average, like random feedback was irrelevant and average performance did not change from no perturbation conditions. The exceptions to this summary were the three conditions that showed consistent error signals (Exp. 1: F1/F2 Coupled Random Perturbation Condition; Exp. 2: Step Condition, Six Condition). In each of these conditions, the error introduced to the feedback was relatively consistent over a span of utterances. This finding is consistent with other indicators that the auditory feedback system eschews correcting the error when the deviation is too large (MacDonald et al., 2010) or if the temporal delay is too great (Mitsuya et al., 2017). Perceptual and motor learning also requires information about the statistics of the environment, and non-stationary environments provide challenges to learning (e.g., Petrov et al., 2006; Narain et al., 2013). When sensory uncertainty exists, it is thought that subjects rely more on their prior estimates of the structure of the task (Körding and Wolpert, 2004). The detection of the uncertainty of the sensory information can be seen as equivalent to the relevance of feedback to performance of a task.

An outstanding issue is whether there is some flexibility in the use of auditory feedback in speech control. Lametti et al. (2012) suggested that individuals prioritized different sources of sensory information. Some people were more influenced by auditory feedback, while others were more reliant on somatosensory signals. In contrast to these individual differences in sensory processing are studies that indicate contextual modification of use of the auditory signal. There are indications that auditory errors can have reduced impact on speech if the signals seem irrelevant [see Wei and Körding (2009) for a study of feedback relevance in limb movements]. Daliri and Dittman (2019) used a ‘clamping’ technique in which the auditory feedback was not contingent on the talker’s productions. The error was constant even when the talker compensated. This ‘irrelevant’ feedback, which was not contingent on the talkers’ behavior, reduced the magnitude of adaptation.

The increase in variability in the Perturbation phases of the current experiments may be indicative of a destabilizing effect of the random perturbations. While our repeated measures designs and the repetitive nature of our protocols are possible explanations as well, within-subject variability did not significantly differ in F1 or F2 in the Control Condition in Experiment 2. However, the heightened variability in the Return phase of Experiment 1 is consistent with this possibility. While we are using the relative variability as a measure of the system’s organization of auditory feedback processing, there are other possible contributions to changes in variability. Bays and Wolpert (2007) review a number of computational ways that the motor system can reduce the unpredictability of sensory information and thus counteract the potentially destabilizing effects of feedback uncertainty. One of these solutions is the integration of multisensory information to improve prediction. The importance of both somatosensory and auditory information in speech motor control is highlighted in theoretical accounts (e.g., Tourville and Guenther, 2011), although the experimental study of dynamic auditory and proprioceptive cues are technically difficult and infrequently attempted (cf., Lametti et al., 2012).

Auditory feedback processing as studied in the laboratory setting has many of the characteristics of phenomena that have driven concerns about the Reliability Paradox (see recent symposium at the Psychonomics Society 2021 meeting). There are a number of phenomena which are robust at a group average level but are not always apparent at the individual subject level (see Nault and Munhall, 2020). Test–retest reliability is also not strong in phenomena that are frequently included in clinical test batteries (e.g., the Stroop test, Implicit Association Test). The lack of robustness at the individual participant level of auditory feedback effects is somewhat unsettling. How can an error-correction system that is supposedly guiding speech motor control be so difficult to demonstrate? One answer is that auditory feedback is not necessary or sufficient for the control of learned speech sequences. Evidence from those who are deafened as adults can be interpreted as supporting this suggestion. While precision of some phonemes degrades, it does so slowly over time and not completely (Cowie et al., 1982). A second answer is that the precision and need for error-based correction of speech is overrated. Fluent speech is a remarkable motor skill, but its required precision is not as high as some manual skills (Uccelli et al., 2021), microsaccades (Poletti et al., 2020) and perhaps less than the bite force requirements of the mandible in chewing. In an analysis of the Switchboard Corpus, Greenberg (1999) reported that significant proportions of phonemes are substituted or deleted in this database. This indicates that intelligibility in communication does not always require the kind of error-correcting precision that the feedback paradigm might suggest.

Another contributing factor in formant-feedback processing is error in measurement (Shadle et al., 2016), particularly in speech produced with higher fundamental frequencies. This problem will have an impact on the data quality but can also have an impact on the quality of the perturbations. In addition to the difficulties associated with formant tracking, the data used to summarize performance makes assumptions about what feedback parameter is important for the talker. It is common, such as was done in the experiments presented here, to use an average formant frequency measured near the midpoint of the vowel. However, talkers may be using other aspects of vowels to control articulation than static indices of formant frequency. Vowels have inherent formant dynamics that vary with dialect, age, and gender of speakers (e.g., Stanley et al., 2021). These dynamics can influence compensatory behavior with participants correcting for changes in spectral trajectories (Jibson, 2020).

Overall, the present results are consistent with a control system that takes into account the statistics of the sensory environment. Two of the conditions point to this conclusion. In Experiment 1, the F1/F2 Coupled Random Perturbation Condition had a mean perturbation value that differed from the baseline value across the 30 trials. This restricted or biased random error signal generated a compensatory response reflecting the average. In Experiment 2, keeping the perturbation constant for six trials also produced differential response from the pattern of responses for shorter perturbations. Our lag correlation analysis in Experiment 2 is also indicative of a control system that is not instantaneously responsive to introduced error. Rather, it appears to be sensitive to the consistency and reliability of the error, integrating information and initiating compensatory behavior over a longer time span. In the context that we are testing, more specific studies focused on the predictability shown in these conditions and how the nervous system computes the consistency are warranted (Burge et al., 2008).

## Data availability statement

The raw data supporting the conclusions of this article are publicly available on OSF here: https://osf.io/n4pgf.

## Ethics statement

The studies involving human participants were reviewed and approved by the Health Sciences Research Ethics Board at Western University (Experiment 1) and the General Research Ethics Board at Queen’s University (Experiment 2). All participants provided their written informed consent prior to participating in these studies.

## Author contributions

DRN collected the data for Experiment 2, performed data analyses for both experiments, and assisted with the writing and editing of this manuscript. TM collected the data for Experiment 1. DWP performed a portion of the data analysis, implemented the experimental system, and helped with manuscript editing. KGM contributed to all aspects of this manuscript. All authors contributed to the article and approved the submitted version.

## Footnotes

1 One complication in our approach is that speech targets are an unknown quantity. Unlike eye-hand coordination, where targets can be experimentally defined and error be measured from a physical target location, speech targets can only be experimentally defined by a linguistic category. Subjects are instructed to say a word or syllable and they select their target. The target and target width can only be inferred from repetitive utterances produced under the same conditions.

2 There is a strategy in several laboratories to instruct subjects to prolong their syllables to study rapid, closed-loop control of speech. While this strategy has been used to increase experimental efficiency and to enable the study of both rapid closed-loop and feed-forward control in the same trials, we are concerned that the prolonged utterances are a different phenomenon than naturally-paced productions. Indeed, it has been suggested that online responses to feedback perturbations and the between-trial effects that we are studying are controlled by different neural mechanisms (Raharjo et al., 2021).

3 We also computed correlations at the individual participant level, and they showed similar trends. Due to space limitations, these correlations were not included in the main text of the manuscript. They are publicly available on OSF here: osf.io/n4pgf.
