# Supplementary material for: Perturbing the consistency of auditory feedback in speech
Source: Front Hum Neurosci. 2022 Aug 25;16:905365. doi: 10.3389/fnhum.2022.905365 (PMC9453207; doi:10.3389/fnhum.2022.905365)
Supplement: Supplementary file 1 [file Data_Sheet_1.docx]

**SUPPLEMENTARY MATERIALS**

|  | **F1/F2 Random** | **F1-Only Random** | **F1/F2 Coupled Random** |
| --- | --- | --- | --- |
| **Mean F1 (SD)** |  |  |  |
| Baseline | 724.15 (4.53) | 720.81 (4.01) | 723.22 (3.23) |
| Perturbation | 719.68 (5.90) | 716.00 (6.86) | 691.33 (8.77) |
| Return | 723.84 (5.86) | 711.97 (4.85) | 710.26 (10.08) |
| **Mean F2 (SD)** |  |  |  |
| Baseline | 2120.29 (12.44) | 2130.15 (10.56) | 2102.72 (7.24) |
| Perturbation | 2122.92 (10.16) | 2116.36 (9.67) | 2141.40 (11.90) |
| Return | 2119.74 (10.44) | 2107.83 (8.37) | 2113.50 (13.12) |

**Table S1.** Between-speaker means and standard deviations (SD) of raw F1 and F2 values in the F1/F2 Random Perturbation condition, F1-Only Random Perturbation condition (B), and F1/F2 Coupled Random Perturbation condition of Experiment 1.

|  | **F1/F2 Random** | **F1-Only Random** | **F1/F2 Coupled Random** |
| --- | --- | --- | --- |
| **Mean F1 (SD)** |  |  |  |
| Baseline | 724.48 (18.29) | 720.79 (21.34) | 723.62 (22.54) |
| Perturbation | 719.66 (23.22) | 715.98 (23.25) | 691.30 (25.75) |
| Return | 723.89 (23.15) | 711.94 (24.76) | 710.20 (25.51) |
| **Mean F2 (SD)** |  |  |  |
| Baseline | 2120.14 (37.86) | 2131.23 (38.46) | 2103.43 (38.33) |
| Perturbation | 2123.19 (40.71) | 2116.40 (38.58) | 2141.59 (40.33) |
| Return | 2119.79 (39.33) | 2108.35 (37.94) | 2113.40 (42.75) |

**Table S2.** Within-speaker means and standard deviations (SD) of raw F1 and F2 values in the F1/F2 Random Perturbation condition, F1-Only Random Perturbation condition (B), and F1/F2 Coupled Random Perturbation condition of Experiment 1

| Condition | Phase (I) Phase (J) | Estimate | Std. Error | *p-*value |
| --- | --- | --- | --- | --- |
|  |  |  |  |  |
| F1/F2 Random | Baseline Perturbation  Return | 4.65  0.46 | 1.80  1.80 | 0.359  1.00 |
|  | Perturbation Return | -4.19 | 1.13 | 0.008 |
| F1-Only Random | Baseline Perturbation  Return | 4.79  8.87 | 1.79  1.79 | 0.273  <.001 |
|  | Perturbation Return | 4.08 | 1.13 | 0.011 |
| F1/F2 Coupled Random | Baseline Perturbation  Return | 32.50  13.46 | 1.79  1.79 | <.001  <.001 |
|  | Perturbation Return | -19.04 | 1.13 | <.001 |

**Table S3.** Pairwise comparisons from the Best Fit Model used to predict speakers’ F1 values during the Baseline, Perturbation, and Return phases of the F1/F2 Random Perturbation condition, F1-Only Random Perturbation condition, and the F1/F2 Coupled Random Perturbation condition of Experiment 1. Significance values were determined using the Bonferroni correction.

| Condition | Phase (I) Phase (J) | Estimate | Std. Error | *p-*value |
| --- | --- | --- | --- | --- |
|  |  |  |  |  |
| F1/F2 Random | Baseline Perturbation  Return | -3.10  0.36 | 2.97  2.97 | 1.000  1.000 |
|  | Perturbation Return | 3.46 | 1.88 | 1.000 |
| F1-Only Random | Baseline Perturbation  Return | 14.66  22.73 | 2.98  2.98 | <.001  <.001 |
|  | Perturbation Return | 8.07 | 1.88 | <.001 |
| F1/F2 Coupled Random | Baseline Perturbation  Return | -37.91  -9.59 | 2.98  2.98 | <.001  0.047 |
|  | Perturbation Return | 28.33 | 1.88 | <.001 |

**Table S4.** Pairwise comparisons from the Best Fit Model used to predict speakers’ F2 values during the Baseline, Perturbation, and Return phases of the F1/F2 Random Perturbation condition, F1-Only Random Perturbation condition, and the F1/F2 Coupled Random Perturbation condition of Experiment 1. Significance values were determined using the Bonferroni correction.

|  | **Control** | **One** | **Three** | **Six** | **Step** |
| --- | --- | --- | --- | --- | --- |
| **Mean F1 (SD)** |  |  |  |  |  |
| Baseline | 731.57 (9.43) | 720.68 (6.15) | 728.19 (8.44) | 726.77 (6.37) | 730.89 (7.82) |
| Perturbation | 727.47 (6.87) | 719.94 (5.97) | 722.91 (12.51) | 720.19 (12.71) | 696.46 (11.89) |
| Return | 723.10 (6.13) | 720.53 (6.33) | 728.51 (7.30) | 721.99 (9.74) | 714.66 (10.96) |
| **Mean F2 (SD)** |  |  |  |  |  |
| Baseline | 2047.54 (9.22) | 2054.44 (11.26) | 2047.88 (12.95) | 2038.36 (9.94) | 2050.93 (12.44) |
| Perturbation | 2045.35 (11.49) | 2039.59 (17.46) | 2038.62 (25.26) | 2041.67 (21.33) | 2093.10 (14.86) |
| Return | 2046.49 (12.79) | 2034.76 (11.82) | 2023.55 (12.17) | 2032.70 (12.03) | 2059.84 (17.67) |

**Table S5.** Between-speaker means and standard deviations (SD) of raw F1 and F2 values in the Control, One, Three, Six, and Step conditions of Experiment 2.

|  | **Control** | **One** | **Three** | **Six** | **Step** |
| --- | --- | --- | --- | --- | --- |
| **Mean F1 (SD)** |  |  |  |  |  |
| Baseline | 732.16 (30.42) | 720.57 (29.78) | 728.03 (27.21) | 726.99 (26.62) | 730.95 (28.32) |
| Perturbation | 727.43 (29.16) | 720.25 (27.96) | 722.79 (33.22) | 720.22 (34.57) | 696.57 (33.30) |
| Return | 723.04 (28.65) | 720.67 (27.73) | 728.39 (30.11) | 720.55 (31.19) | 714.42 (31.07) |
| **Mean F2 (SD)** |  |  |  |  |  |
| Baseline | 2046.85 (44.67) | 2053.63 (50.02) | 2048.06 (42.76) | 2038.63 (41.24) | 2050.32 (46.88) |
| Perturbation | 2046.07 (47.58) | 2036.99 (51.21) | 2037.59 (54.68) | 2041.33 (54.85) | 2092.59 (48.86) |
| Return | 2045.47 (48.78) | 2034.32 (46.29) | 2023.92 (47.16) | 2032.05 (47.21) | 2059.46 (53.48) |

**Table S6.** Within-speaker means and standard deviations (SD) of raw F1 and F2 values in the Control, One, Three, Six, and Step conditions of Experiment 2.

| Condition | Phase (I) Phase (J) | Estimate | Std. Error | *p-*value |
| --- | --- | --- | --- | --- |
|  |  |  |  |  |
| Control | Baseline Perturbation  Return | 4.45  8.95 | 2.66  2.66 | 1.000  0.080 |
|  | Perturbation Return | 4.50 | 2.16 | 1.000 |
| One | Baseline Perturbation  Return | 0.44  -0.08 | 2.64  2.64 | 1.000  1.000 |
|  | Perturbation Return | -0.52 | 2.16 | 1.000 |
| Three | Baseline Perturbation  Return | 5.25  -0.37 | 2.64  2.65 | 1.000  1.000 |
|  | Perturbation Return | -5.62 | 2.16 | 0.992 |
| Six | Baseline Perturbation  Return | 6.61  5.20 | 2.65  2.65 | 1.000  1.000 |
|  | Perturbation Return | -1.41 | 2.16 | 1.000 |
| Step | Baseline Perturbation  Return | 34.18  16.25 | 2.66  2.66 | <.001  <.001 |
|  | Perturbation Return | -17.94 | 2.16 | <.001 |

**Table S7.** Pairwise comparisons from the Best Fit Model used to predict speakers’ F1 values during the Baseline, Perturbation, and Return phases of the Control, One, Three, Six, and Step conditions of Experiment 2. Significance values were determined using the Bonferroni correction.

| Condition | Phase (I) Phase (J) | Estimate | Std. Error | *p-*value |
| --- | --- | --- | --- | --- |
|  |  |  |  |  |
| Control | Baseline Perturbation  Return | 1.60  1.86 | 4.03  4.04 | 1.000  1.000 |
|  | Perturbation Return | 0.26 | 3.30 | 1.000 |
| One | Baseline Perturbation  Return | 16.74  19.75 | 4.02  4.01 | 0.003  <.001 |
|  | Perturbation Return | 3.01 | 3.29 | 1.000 |
| Three | Baseline Perturbation  Return | 10.92  24.00 | 4.05  4.04 | 0.729  <.001 |
|  | Perturbation Return | 13.08 | 3.29 | 0.007 |
| Six | Baseline Perturbation  Return | -2.66  6.84 | 4.03  4.03 | 1.000  1.000 |
|  | Perturbation Return | 9.50 | 3.29 | 0.405 |
| Step | Baseline Perturbation  Return | -42.65  -9.48 | 4.03  4.04 | <.001  1.000 |
|  | Perturbation Return | 33.17 | 3.28 | <.001 |

**Table S8.** Pairwise comparisons from the Best Fit Model used to predict speakers’ F2 values during the Baseline, Perturbation, and Return phases of the Control, One, Three, Six, and Step conditions of Experiment 2. Significance values were determined using the Bonferroni correction.
